# Supplementary material for: Deletion of an sRNA primes development in a multicellular bacterium
Source: iScience. 2025 Feb 12;28(3):111980. doi: 10.1016/j.isci.2025.111980 (PMC11928866; doi:10.1016/j.isci.2025.111980)
Supplement: Document S1. Figures S1–S7 [file mmc1.pdf]

iScience, Volume *28*

Supplemental information

Deletion of an sRNA primes development in a multicellular bacterium

Marco La Fortezza, Jasper Verwilt, Sarah M. Cossey, Sabrina A. Eisner, Gregory J. Velicer, and Yuen-Tsu N. Yu

Supplementary Figures

7 Supplementary Figures

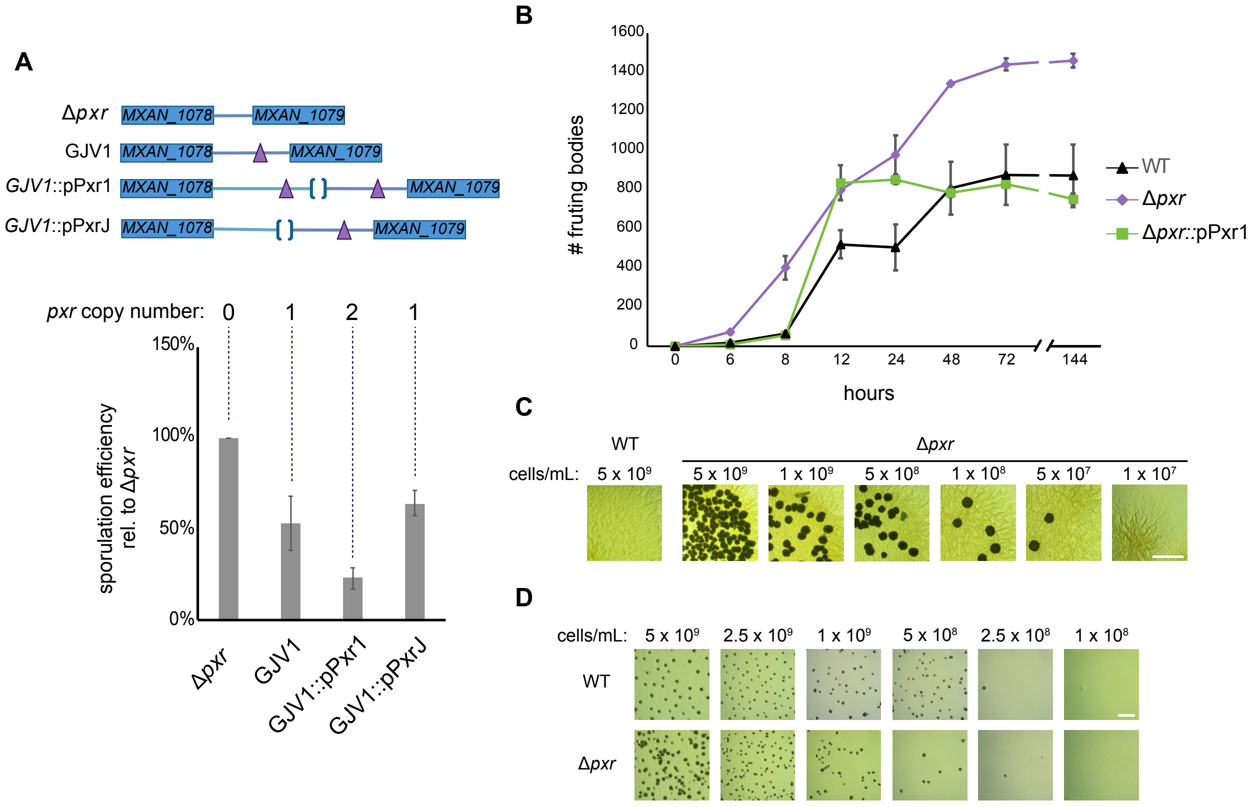

Supp. Fig. 1. *pxr* gene-copy number and expression influence sporulation efficiency, fruiting-body formation, and cellular density in *Myxococcus xanthus.* A) Quantification of the sporulation efficiency as a function of *pxr* gene-copy number. Increasing the number of *pxr* copies reduces sporulation efficiency on starvation plates, reflecting negative regulation by Pxr. The genetic layout of each strain is diagramed on the upper panel. In the diagram, purple triangles represent the *pxr* gene, the light blue line indicates the integrated genetic region (carrying a *pxr* locus or not), and the brackets represent the plasmid region inserted into the *pxr* native site. The bar chart below shows the average sporulation efficiency – relative to the *Δpxr* strain – of three strains bearing either one or two copies of *pxr***.** Error bars indicate the standard error calculated from three independent biological replicates. **B)** Plot reporting the average number of fruiting-body counts over time for WT (black line and triangles), *Δpxr* (purple line and diamonds), and *Δpxr::pPxr1* with restored *pxr* expression. The error bars represent the standard errors associated with each measurement (n = 3). **C**) Microscopy images comparing five-day-old WT and *Δpxr* cell cultures inoculated on an agar plate with 0.3% casitone, with decreasing cellular densities for the *Δpxr* mutant. Dark spots are mature fruiting bodies. **D**) Microscopy images showing five-day-old cultures of WT and *Δpxr* (top and bottom array rows, respectively) after inoculation onto buffered (TPM) agar at six different initial densities. Dark spots are mature fruiting bodies. Scale bars equal to 2mm for both panels.

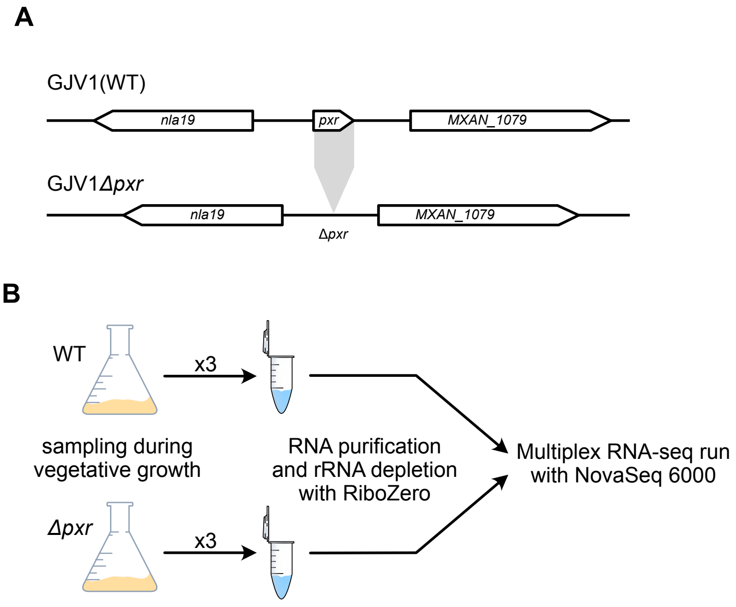

Supp. Fig. 2. Schematic of the *pxr* locus in WT and *Δpxr* mutant cells and experimental design for rna sequencing. A) Schematic representation of the *pxr* locus in WT (GJV1) cells (top) and GJV1*Δpxr* mutant cells (bottom). **B)** Cartoon reporting the experimental design used to sample RNA from cells during vegetative growth in rich media. RNA was extracted, purified, and rRNA removed using RiboZero before whole transcriptome sequencing on a NovaSeq 6000 machine. The total number of reads per sample was estimated to be approximately eight million (total number of annotated genes for DK1622 = 7579).

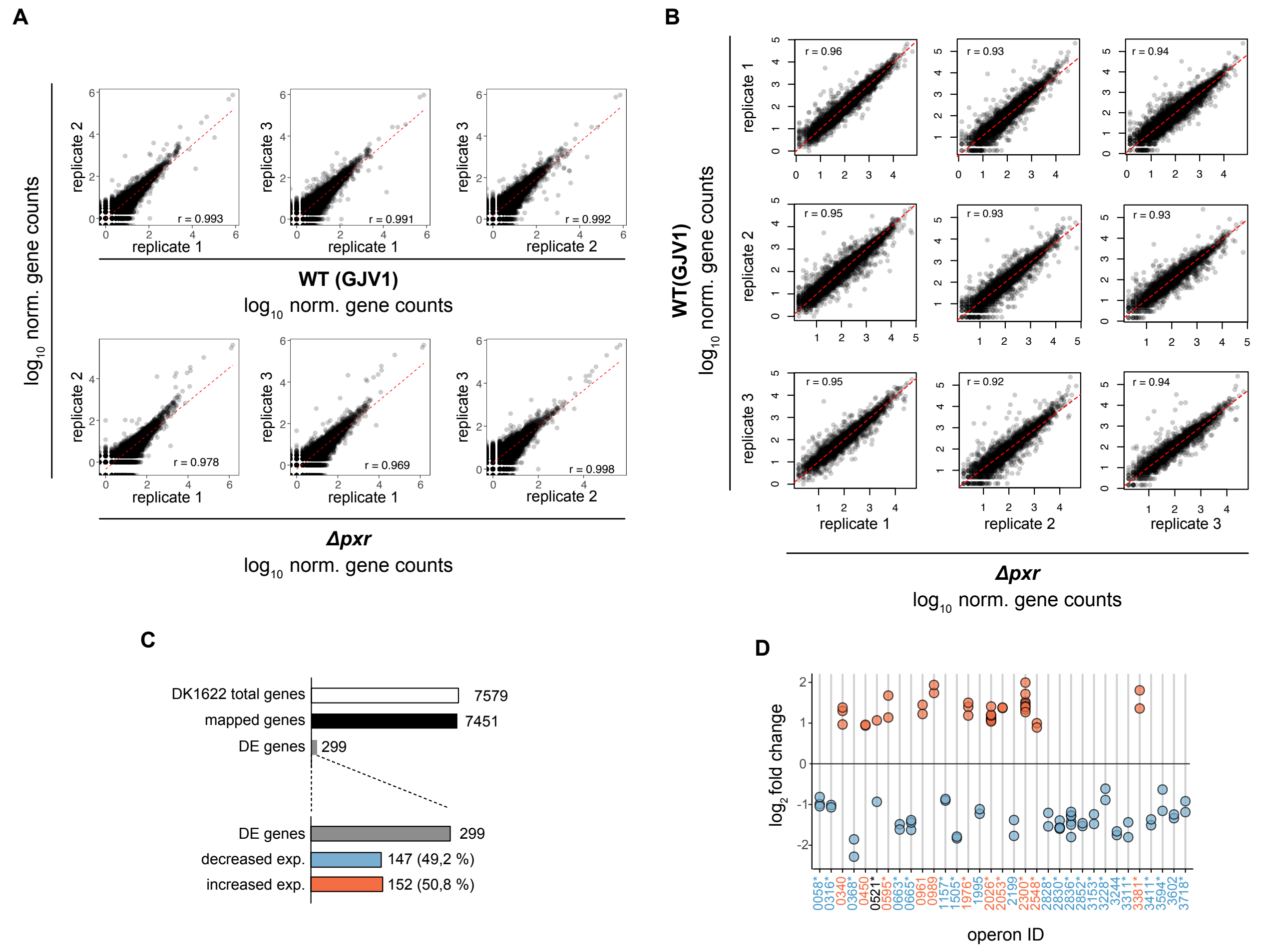

Supp. Fig. 3. Differential gene expression and correlation analysis of WT and *Δpxr* transcriptomes. A) Scatter plots reporting the pairwise comparison of gene counts between the individual biological replicates per each strain (WT or *Δpxr*). Pearson's coefficients of correlation (*r*) are reported within each plot. We measured an average variance value of 0.048 and 0.142 for WT and *Δpxr,* respectively. **B)** Scatter plots reporting the pairwise comparison of gene counts between WT (GJV1) and *Δpxr* individual biological replicates. Pearson's coefficients of correlation (*r*) are reported within each plot. **C)** Top: Summary of the total number of unique transcript sequences mapped onto the DK1622 reference genome (white and black bars, respectively), the number of genes differentially expressed (DE) between WT and the *Δpxr* mutant (grey bars) compared to the mapped and total genes. Bottom: Numbers (and percentages) of DE genes (grey bar) that are down-regulated (blue bar) and up-regulated (red bar). **D)** Dot plot illustrating the level of differential expression of genes (*y* axis) belonging to the list of the enriched operons (*x* axis). The blue and red text refers to operons containing genes with decreased and increased transcript levels. *0521* (in black) is the only operon that contains genes with different signs of expression levels. Asterisks placed next to the operon IDs indicate operons that contain at least one gene associated with development (75% of the 33 operons); see Supp. Table 2 and 5 for more details.

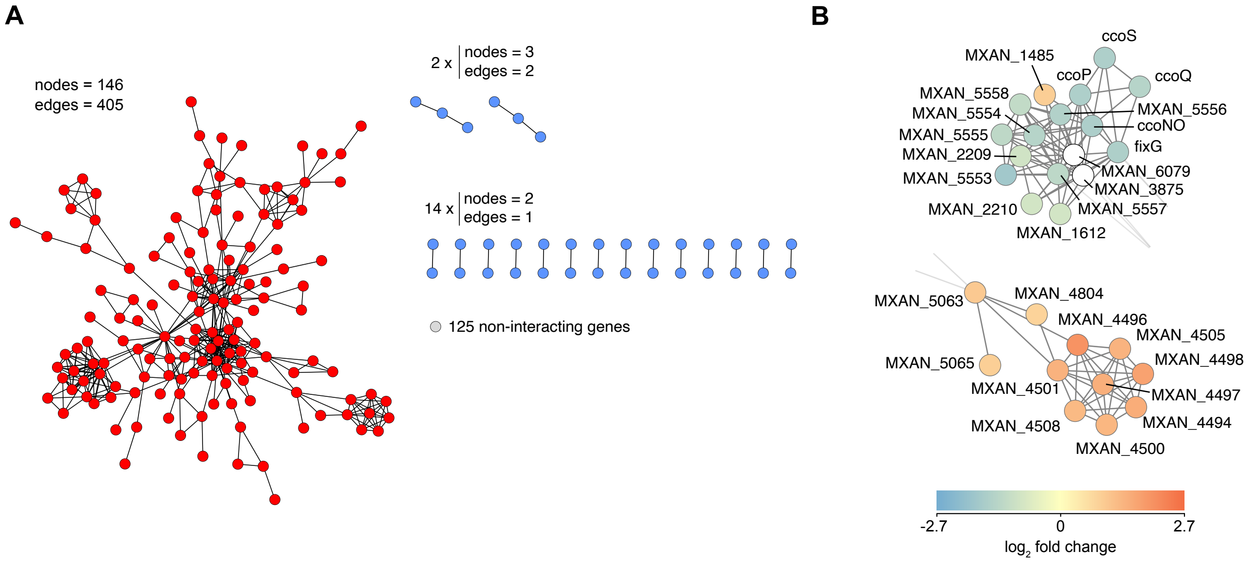

Supp. Fig. 4. Gene-interaction networks and differential expression patterns in WT and *Δpxr* cells A) Summary of all the networks resulting from previously annotated gene interactions. The main network is red, blue which all interactions are less than five genes, and grey, which are all single non-interacting genes. **B)** Close-ups of two dense sub-clusters from the main red network reported in (**A**). Nodes are coloured by their differential expression levels, going from blue (low transcript levels) to red (high transcript levels). The depicted networks can be downloaded and consulted in greater detail online at this **link**.

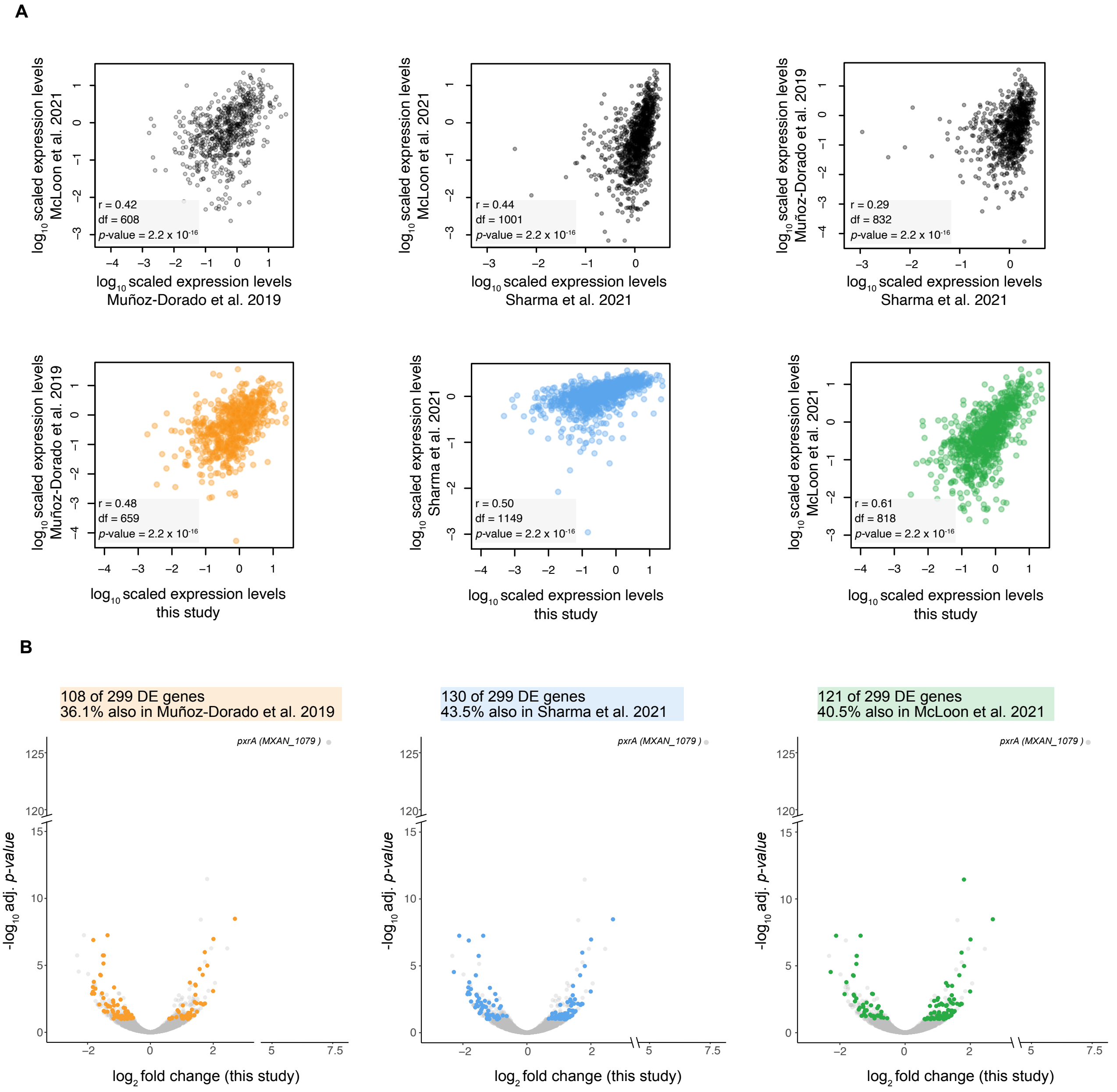

Supp. Fig. 5. Comparative transcriptomic analyses of vegetative cells across multiple studies. A) Top row: three scatter plots with pair-wise contrasts of transcript levels from vegetative cells across the three studies we used to identify developmental genes. Bottom row: three scatter plots contrast the transcript levels of wild-type vegetative cells obtained in our study vs Muñoz-Dorado *et al.* 2019 (orange dots), Sharma *et al.* 2021 (blue dots) and McLoon *et al.* 2021 (green dots). In all scatter plots (top and bottom rows), the summary statistic of the Pearson correlation test (*r*) are reported within each graph (df = degrees of freedom). **B)** Volcano plots reporting differentially expressed (DE) genes found in our study that were previously associated with development by Muñoz-Dorado et al. 2019 (orange dots), Sharma et al. 2021 (blue dots) and/or McLoon et al. 2021 (green dots). A summary of statistics for each comparison is reported above each graph. For more details on comparing RNA-seq profiles, consult Methods*: Comparisons of RNA-seq profiles.* Visit the following link to explore the RNA-seq data in more detail.

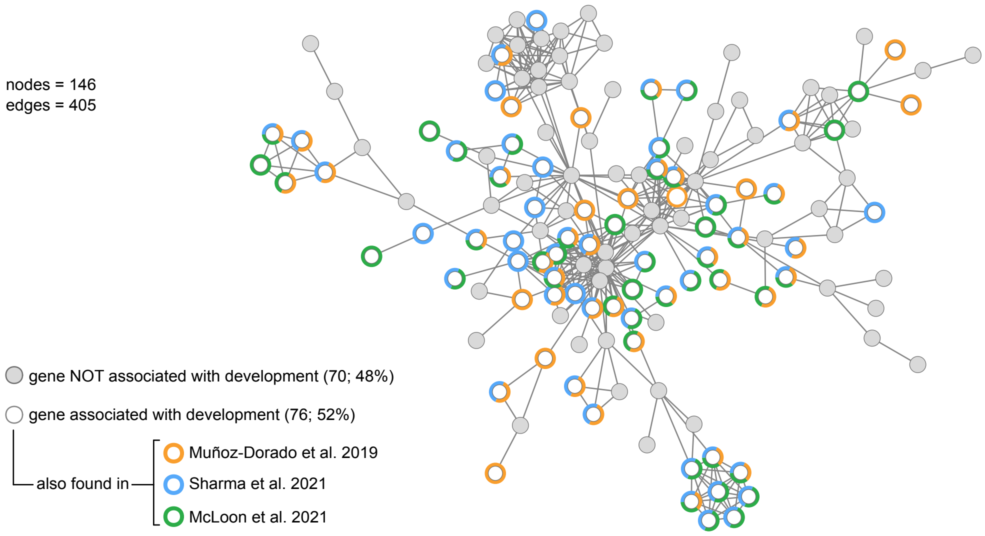

Supp. Fig. 6. Developmental gene mapping within the main interaction network across multiple studies. The main gene interaction network (also in Fig. 2D- and Supp. Fig. 4A) highlights developmental genes in white and genes not associated with development in grey. Coloured circles identify developmental genes present in Muñoz-Dorado et al. 2019 (orange circles), Sharma et al. 2021 (blue circles), and/or McLoon et al. 2021 (green circles). The depicted network can be downloaded and visualised in greater detail using this **link**.

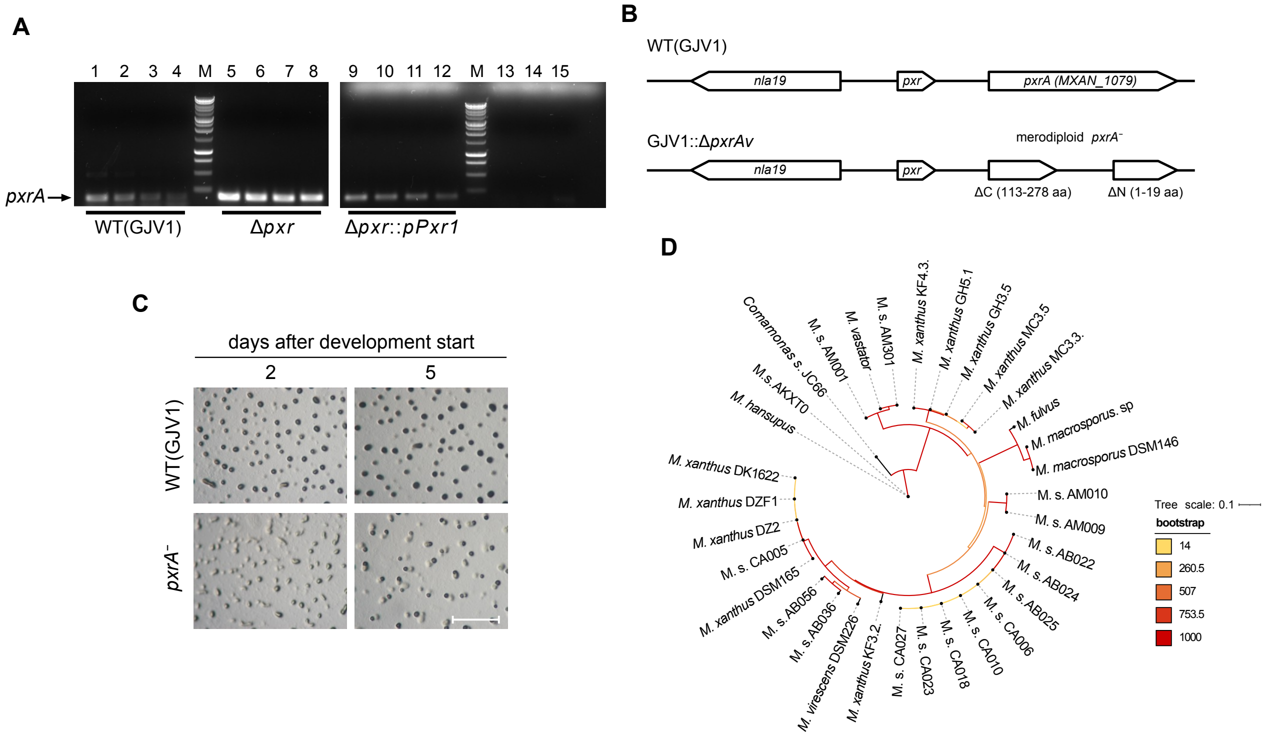

Supp. Fig. 7. Validation of *pxrA* expression, genetic and phenotypic analysis of *pxrA* mutants, and phylogenetic relationships of *pxrA* orthologues. A) RT-PCR analysis showing the relative expression of the *pxrA* gene from the DNase-treated RNA samples derived from WT (GJV1) (lanes 1-4), *Δpxr* (lanes 5-8), and *Δpxr::pPxr1,* which is complemented for *pxr* expression (lanes 9-12). The RT-PCR signals of lanes 1, 5 and 9 were performed from the undiluted RT-treated samples, lanes 2, 6 and 10 from the factor-of-2-diluted samples, lanes 3, 7 and 11 from the factor-of-4-diluted samples and finally, lanes 4, 8 and 12 from the factor-of-8-diluted samples. The same PCR procedure was also performed on the same RNA samples that did not undergo the reverse transcription (RT) step (lane 13: WT(GJV1); lane 14: *Δpxr*, and lane 15: *Δpxr::pPxr1*). These three last non-RT lanes control for the potential presence of signals due to genomic DNA contamination during RNA sample preparation. M indicates the 1 kb DNA ladder (Promega). The arrow indicates the ~180 bp *pxrA* PCR product expected from the RNA transcripts of the RT-PCR assays. **B)** Illustration of the *pxrA* locus in WT(GJV1) and $pxrA^-$ (GJV1*pxrA::pCR-1079*) mutant cells. Merodiploid mutant cells carried the two deletions at the N- (1-19) and C-terminal (113-278) regions of PxrA (total protein length 278 aa). **C)** Representative microscopy images showing the wild-type WT(GJV1) (top row) and the mutant $pxrA^-$ during starvation-induced development. The photographs were taken two and five days after the onset of development. The fruiting bodies are visible as darkened aggregates. The scale bar equals 1 mm. **D)** Phylogenetic tree for PxrA orthologues. Branches are coloured by their bootstrap values, and their lengths indicate the expected number of substitutions per site. The species name and strain are indicated at the end of each branch. M.s. = *Myxococcus sp.*
